# Blended Care-Cognitive Behavioral Therapy for Depression and Anxiety in Real-World Settings: Pragmatic Retrospective Study

**DOI:** 10.2196/18723

**Published:** 2020-07-06

**Authors:** Anita Lungu, Janie Jihee Jun, Okhtay Azarmanesh, Yan Leykin, Connie E-Jean Chen

**Affiliations:** 1 Lyra Health Burlingame, CA United States; 2 Department of Psychology Palo Alto University Palo Alto, CA United States

**Keywords:** cognitive behavior therapy, blended psychotherapy, dissemination, implementation, depression, anxiety/anxiety disorders, internet, web based, video psychotherapy

## Abstract

**Background:**

The past few decades saw considerable advances in research and dissemination of evidence-based psychotherapies, yet available treatment resources are not able to meet the high need for care for individuals suffering from depression or anxiety. Blended care psychotherapy, which combines the strengths of therapist-led and internet interventions, can narrow this gap and be clinically effective and efficient, but has rarely been evaluated outside of controlled research settings.

**Objective:**

This study evaluated the effectiveness of a blended care intervention (video-based cognitive behavior therapy and internet intervention) under real-world conditions.

**Methods:**

This is a pragmatic retrospective cohort analysis of 385 participants with clinical range depression and/or anxiety symptoms at baseline, measured using Patient Health Questionnaire-9 (PHQ-9) and Generalized Anxiety Disorder-7 (GAD-7), who enrolled in blended care psychotherapy treatment. Participants resided in the United States and had access to the blended care intervention as a mental health benefit offered through their employers. Levels of depression and anxiety were tracked throughout treatment. Hierarchical linear modeling was used to examine the change in symptoms over time. The effects of age, gender, and providers on participants’ symptom change trajectories were also evaluated. Paired sample t-tests were also conducted, and rates of positive clinical change and clinically significant improvement were calculated.

**Results:**

The average depression and anxiety symptoms at 6 weeks after the start of treatment were 5.94 and 6.57, respectively. There were significant linear effects of time on both symptoms of depression and anxiety (β=–.49, *P*<.001 and β=–.64, *P*<.001). The quadratic effect was also significant for both symptoms of depression and anxiety (β=.04, P<.001 for both), suggesting a decelerated decrease in symptoms over time. Approximately 73% (n=283) of all 385 participants demonstrated reliable improvement, and 83% (n=319) recovered on either the PHQ-9 or GAD-7 measures. Large effect sizes were observed on both symptoms of depression (Cohen d=1.08) and of anxiety (d=1.33).

**Conclusions:**

Video blended care cognitive behavioral therapy interventions can be effective and efficient in treating symptoms of depression and anxiety in real-world conditions. Future research should investigate the differential and interactive contribution of the therapist-led and digital components of care to patient outcomes to optimize care.

## Introduction

Depression and anxiety are the leading causes of disability worldwide [1]. Within work environments, depression and anxiety have been associated with decreased productivity for employees through absenteeism, presenteeism, increased disability leave, or sick leave [2-4]. Although effective psychological treatments for depression and anxiety exist, they are difficult to access for many individuals [5,6]. Approximately half of the individuals meeting criteria for major depressive disorder in a given year remain untreated or under-treated [7]. Cognitive behavior therapy (CBT) has been rigorously tested and consistently shown to be efficacious in randomized controlled trials and effective in real-world applications for treating depression and anxiety, emerging as the initial treatment of choice [8]. Barriers to large scale dissemination of CBT include insufficient numbers of specialized mental health providers [9], long waitlists to see a provider [10], high cost of therapist delivered care, the stigma associated with seeing a therapist and receiving treatment for mental health disorders [11], as well as travel time required to see a provider in person.

Internet-administered automated cognitive behavior therapy (iCBT) has emerged as a promising cost-effective solution that could narrow the gap between clinical demand and availability of CBT for adults, adolescents, and children [12,13]. iCBT involves the delivery of clinical CBT content via the internet and can involve different formats such as text, video, audio files, and interactive elements. iCBT interventions can be delivered alone or with additional support and guidance from a therapist or a coach [14]. Among the advantages of iCBT over traditional therapist-delivered care are scalability, guaranteed treatment fidelity, increased geographical reach, full temporal availability, ability to progress at one’s own pace, savings in travel time, and cost-effectiveness. Several meta-analyses have found iCBT interventions to be clinically effective for a wide range of clinical disorders, including depression, anxiety disorders, and eating disorders [12,15].

iCBT interventions with human support lead to better clinical outcomes compared to unsupported ones [16]. Results of iCBT interventions with human support are sustained in the long term, and supported interventions have higher adherence rates compared to unsupported ones [12,16]. For unsupported interventions, lack of support to sustain motivation for change and of accountability towards a professional can lead to decreased clinical efficacy and higher dropout rates [17,18]. Many iCBT interventions are relatively static and lack the sophistication to be able to adapt to the client’s initial and evolving clinical presentation.

Blended care CBT treatments (BC-CBT) have emerged as a newer approach that integrates regular therapist-led CBT sessions with iCBT modules into an integrated treatment, taking advantage of the benefits of both approaches while mitigating their disadvantages. Blended interventions can take place with therapist-led sessions delivered in a face-to-face format [19] or via video [20]. Given that part of the treatment is taking place via Internet-based modules, BC-CBT has the potential to decrease the number of sessions with therapists while achieving similar outcomes to therapist-only treatments [21], which can improve the clinical efficiency and scalability of treatment. BC-CBT may also lead to faster improvements when weekly therapist-led sessions are supplemented with iCBT, resulting in a more intensive therapy experience [22]. BC-CBT addresses the fundamental disadvantages for iCBT, specifically the low initial engagement and high dropout rates, through its human component, by enabling the development of a therapeutic alliance found to be associated with higher motivation to initiate and sustain engagement in care [23,24]. Importantly, having the therapist in charge of clinical assessment, treatment plan, and delivery in BC-CBT allows for more personalization of care compared to iCBT. Specifically, the therapist selects digital modules most relevant to the client’s presenting concerns and explains the rationale to the client, linking digital tools to the client’s goals, thereby increasing motivation and compliance to treatment. BC-CBT treatments are clinically efficacious in controlled studies for multiple mental health conditions such as depression, anxiety, and substance abuse [19] and multiple settings such as primary care [25], specialized mental health clinics [26], and inpatient [27]. The vast majority of BC-CBT applications described to date consist of in-person face-to-face therapy sessions supplemented with iCBT modules. However, by moving face-to-face BC-CBT therapy sessions to the telehealth modality (ie, the delivery of care via video or audio), the reach of the treatment can be extended, offering treatment to more individuals in need. It has consistently been found that telehealth mental health services are just as effective as in-person care [28].

To date, there have been very few large-scale studies examining the effectiveness of BC-CBT interventions delivered via telehealth on a large scale in real-world settings [20,29]. Dissemination of most interventions outside of tightly controlled research environments introduces challenges, including therapist drift, potentially more complex clinical presentations, as well as possible lower treatment engagement [30], and BC-CBT would likely face similar challenges. Additionally, for BC-CBT delivered by telehealth, acceptability of the video communication channel, as well as the iCBT care components by both therapists and clients, are likely necessary to prevent dropout and preserve clinical effectiveness.

The present study uses data gathered as part of routine care for clients who received BC-CBT treatment as a benefit offered through their employer. The BC-CBT program combines live video-based sessions with a therapist with technology and evidence-based care tools for clients to use in between sessions. BC-CBT therapists are supported in their work to implement BC-CBT via regular individual and group consultations; clients have access to and are encouraged to use evidence-based care tools at any time. These elements can make the psychotherapy experience more clinically efficient, enabling more clients to improve faster. To our knowledge, to date, no study in the United States has examined BC-CBT interventions at a large scale under real-world conditions.

## Methods

### Study Design

This pragmatic retrospective cohort study used data collected for quality control of a BC-CBT program. Participants resided in the United States and had access to the BC-CBT program as a mental health benefit at no cost to them from their employer companies. Lyra Health partners with Lyra Clinical Associates to offer a behavioral health benefit to companies through which employees and dependents have access to a video BC-CBT program. Employees and their dependents learned of this mental health benefit through information from their employer and could then access the benefit through registering online with Lyra Health, searching for a provider, and directly enrolling in the BC-CBT program. The program’s rigorous quality assurance elements included ongoing individual and team-based clinical consultations and clinical case reviews informed by routine outcome monitoring of depression and anxiety symptomatology.

All participants who engaged in the BC-CBT program were asked to complete electronically secure, standardized measures of depression and anxiety every week as well as a satisfaction measure at the end of treatment. No specific length of care was defined. Participants had access to a minimum of 12 sessions, depending on the benefit offered by the sponsoring company. This post-factum analysis of deidentified data gathered from treatment offered by Lyra Clinical Associates was determined to be not human subject research by the Palo Alto University Institutional Review Board.

### Participants and Data Inclusion

Participants were individuals who started BC-CBT treatment between November 1, 2018, and September 1, 2019. Participants who sought psychotherapy went through an online onboarding process and answered questions about their symptoms, the impact of symptoms on their general functioning, and their interest in receiving care via video. Those who were not open to seeing a provider via video were referred to in-person therapy and not offered BC-CBT. Exclusion criteria for the BC-CBT program were: being under 18 years of age, active suicidality/self-harm, active homicidality, a current diagnosis of a mental health disorder with psychotic features not stabilized on medications, unstable bipolar disorder, and a current diagnosis of severe alcohol or substance use disorder. Participants also had to score above the clinical cut-off for either the Patient Health Questionnaire-9 (PHQ-9≥10) or the Generalized Anxiety Disorder-7 (GAD-7≥8) on a baseline assessment (n=555). No additional diagnostic assessment was conducted for the study, though each therapist conducted independent clinical interviews for treatment purposes. We considered a baseline assessment to be invalid if it was collected more than 2 weeks before the first therapy session or after the second therapy session. We excluded 30 records with invalid baselines from analyses based on this definition. Participants for whom no assessment was available within 5 weeks after the last therapy session were excluded. We excluded from the analysis 140 records with invalid second assessments per this definition, leaving 385 participant records for analysis. The final scores were the last valid available datapoint after baseline.

**Figure 1:**
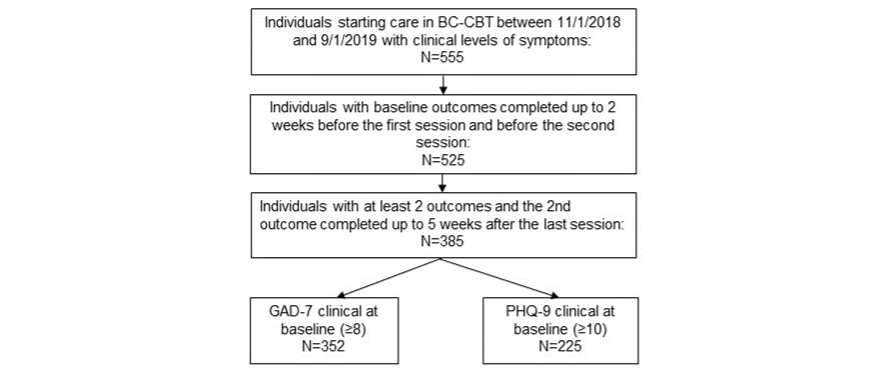
Participant flow.

For two-thirds of the included records (63%, n=242), final scores were collected after the final therapy session (but <5 weeks after the final session), and the remainder was collected from an earlier session (Figure 1).

### Self-Report Measures

Assessments consisted of the PHQ-9 [31] and the GAD-7 [32], well-validated measures of depression and anxiety, respectively. Clinical cut-offs were PHQ-9≥10 and GAD-7≥8, as research suggests that participants who score at or above these cut-offs are very likely to meet criteria for major depression [31] or to have an anxiety disorder diagnosis [33].

### Treatment

The BC-CBT program combined live video-based sessions with a therapist plus digital care tools (lessons and exercises) that clients could access and were encouraged to use between sessions (Multimedia Appendix 1). The digital therapy platform supporting BC-CBT had separate portals with customized functions for clients and therapists, respectively.

#### Individual Video Psychotherapy

Therapy sessions were conducted on a secure, proprietary HIPAA-compliant video platform developed by Lyra Health. Therapy staff consisted of 49 licensed therapists (licensed clinical psychologists, licensed marriage and family therapists, licensed clinical social workers, or licensed professional counselors) vetted for their commitment to and proficiency in CBT via extensive application reviews and clinical interviews. Approximately 41% (n=20) of the therapists had less than 5 years of experience, 32% (n=16) had between 5 and 10 years of experience, and 27% (n=13) had over 10 years of experience. All therapists were trained on-site with a 2-day workshop on BC-CBT. The mean number of clinical cases per therapist was 8.2 (SD 7.1). Participants engaged in BC-CBT met with their therapists, either weekly or bi-weekly. A core focus of the program was to ensure a high quality of evidence-based clinical care via providing therapists with peer supervision. Providing clinicians with ongoing consultation and support has been identified as a critical component of treatment dissemination [34] that helps maintain and enhance clinicians’ skills [35,36]. Sessions were recorded with the participant’s consent. Quality adherence of clinical care was ensured via session review, bi-weekly individual consultation video calls, and video consultation groups with other licensed therapists. The weekly assessment of depression and anxiety symptoms represented important information helping to focus the quality assurance process on clients who were not making progress as expected.

#### Digital Lessons and Exercises

Following each session, therapists assigned educational digital lessons and exercises to be completed by clients before their next session. The digital treatment components were developed by Lyra Health based on transdiagnostic treatment approaches, such as the Unified Treatment Protocol [37], Acceptance Commitment Therapy [38], and Dialectical Behavior Therapy [39,40]. Example principles and skills taught in the digital lessons and exercises included: clarifying values, understanding emotions, mindful awareness, cognitive restructuring, challenging avoidance (via behavioral activation and exposure), and communication skills.

The digital lessons consisted of animated videos and quizzes to test comprehension and provide corrective feedback. The digital lessons utilized a storytelling approach wherein viewers followed the therapy journey for characters presenting with symptoms of depression or anxiety. This approach has been used in other efficacious iCBT interventions [41] and found to have a normalizing effect for clients. Therapists personalized digital tools in several ways. First, therapists selected digital lessons and exercises to match clients’ case formulation (clinician’s understanding of clients’ presenting concerns viewed from a CBT perspective). Second, to increase motivation for completion of the personalized digital tools, therapists introduced them by linking them to issues immediately relevant to the client and discussed during that specific session, as well as to clients’ broader goals. Third, therapists received alerts when clients completed assigned digital lessons and exercises and could send personalized feedback to clients.

#### The BC-CBT Therapy Platform for Providers

The BC-CBT platform enabled therapists to conduct a variety of BC-CBT therapy tasks before, during, after, and in between therapy sessions seamlessly in a single environment.

Before therapy sessions, therapists could check whether clients had submitted PHQ-9 and GAD-7 outcomes to track their progress in treatment. They could also review clients’ completion of assigned practices, clinical outcomes, and communicate asynchronously with clients via secure messaging to encourage them or support them in applying therapy principles and skills.

Therapists utilized the platform to hold their video sessions in a HIPAA secure environment and to record sessions for quality assurance with clients’ consent. During sessions, therapists could also share their screen to collaboratively complete digital exercises with the client or preview specific exercises and digital lessons they intended to assign to the client. Modeling such activities was done to increase the likelihood that the client would later complete the exercise or lesson on their own. Providing users with the ability to perform a “virtual rehearsal” of a desired new behavior within a technology tool (such as completing a psychotherapy digital exercise) has been proposed as a feature that can change behavior in the real world [42]. Screen sharing also allowed therapists to review the clinical outcomes responses and progress with their clients, which has been found to make treatment more efficacious and efficient [43].

After sessions, therapists completed therapy notes in the BC-CBT platform. The notes were designed as a decision support system [42] to a) help clinicians create case conceptualizations for clients that would guide treatment goals and treatment plans, b) ensure that treatment goals were indeed addressed during session time, c) increase adherence to the digital elements of BC-CBT like assigning and reviewing digital lessons and exercises, and d) increase consistency and thoroughness of conducting more complex clinical tasks, such as safety risk assessments.

#### The BC-CBT Therapy Platform for Clients

The digital platform behind BC-CBT supported clients in completing clinical outcomes as well as assigned digital lessons and exercises in several ways. First, clients received automatic notifications encouraging them to complete the assigned outcome measures and digital lessons and exercises if they had not already, and a new session was approaching. Second, clients were also alerted if their therapist sent them feedback on a completed exercise. Third, “tunneling” was utilized as a digital persuasion technique to increase the completion of digital assignments [42]. For example, the client was guided through a digital lesson composed of multiple parts (animated videos, comprehension quiz) such that subsequent parts automatically started if the client did not explicitly stop the experience. Last, clients could revisit all digital lessons and materials assigned for the duration of treatment.

### Data Analyses

Two analytical methods were employed to estimate the impact of BC-CBT on depression and anxiety symptomatology. Hierarchical linear modeling (HLM) was used to examine change over time. HLM is a statistical technique that is capable of modeling incomplete data over time. The routinely administered outcomes measures were included as weekly observations for participants. In cases when participants completed multiple outcomes measures during the same week in treatment, an average of the scores for that week was calculated. We included all participants who provided at least two outcome measures meeting all other inclusion criteria. In order to estimate the change in outcomes, we tested for a linear effect of time and a quadratic effect of time, modeling the slowing of the rate of change. HLM estimates the individual slopes and intercepts for all participants, as well as the sample’s average slope and intercept. Participants’ age, gender, and provider were added to the analysis as level-2 predictors of the intercept, slope, and quadratic component. For both dependent variables we added level 2 predictors, age, gender, provider, at the intercept, slope, and quadratic effect. As a supplementary statistical analysis, we conducted paired samples *t*-tests between baseline and last available assessment scores for each measure. We calculated Cohen *d*, a conservative measure of effect size for within-subjects designs that controls for the correlation between measurements. We then assessed whether participants attained reliable clinical change (a measure of symptom change beyond what could be attributed to measure error alone), recovery (moving from the clinical range to the subclinical range), and clinically significant change (demonstrated reliable change and recovery; [44]) on either the PHQ-9 or GAD-7.

We used the Chi-square test to assess differences in the distribution of genders between records with a valid baseline assessment that were not included in the analyses (n=140) and those that were included (n=385). Independent samples *t*-tests were used to evaluate differences between those groups in age as well as baseline depression and anxiety symptoms scores. To provide more detailed information on rates of improvement, we performed the paired samples t-test and reliable clinical change and recovery analyses for the entire sample of clients but also separately for individuals scoring in the clinical range of symptoms for depression, anxiety, or both.

## Results

### Overview

There were no statistically significant differences between records with a valid baseline assessment versus those that were not included in the analyses by gender (χ^2^_2_=1.13, *P*=.56), age (t_523_=1.08, *P*=.28), baseline depression severity (t_523_=0.98, *P*=.33) and baseline anxiety severity (t_523_=0.86, *P*=.39).

Participants’ mean age was 32.8 years (SD 8.0). The majority of participants were between 25 and 34 years old (55%; n=212), 25.4%% (n=98) between 35 and 44 years old, 11.2% (n=43) were between 18 and 24 years old, and 8.3% (32) were above 45 years old. The majority of participants were women (63.4%; n=244), 35.8% (n=138) were men, and 0.8% (n=3) did not specify their gender. The mean number of sessions delivered during a course of BC-CBT was 5.2 (SD 2.9; range 1-17). The mean number of weeks participants spent in treatment was 6.4 (SD 5.3; range 1-25).

### Hierarchical Linear Models

Based on prior work that found a negatively accelerated relationship between the number of therapy sessions and improvement in care [45] as well as the observed sample mean in our dataset, we fitted an HLM with intercept, slope, and quadratic effect of time. The time variable was centered at the point of the average length of care (week 6) to reduce collinearity between the linear and quadratic components while keeping the interpretation of the model’s parameters more meaningful [46].

Likelihood ratio testing revealed that a quadratic effect of time provided a significant improvement in model fit over a simple linear effect of time. A cubic effect of time was also attempted but found not to improve model fit significantly. The results of the model indicate that all growth effects were significant (Table 1). Specifically, the slopes were negative, suggesting that symptoms of depression and anxiety decrease with time in care. The quadratic slope effects were positive, suggesting that rapid progress initially in care tapers off as clients advance in treatment. There was also significant variability in the growth parameters, indicating that there were significant differences between individuals in how participant’s depression and anxiety symptoms changed during care. Likelihood ratio testing revealed a better fit for models allowing both the linear and quadratic components to vary. In other words, participants varied in the trajectory and rate of change in treatment.

For depression symptoms, at 6 weeks after starting treatment (which represents the average length of care), the mean level of symptoms in the sample was 5.94. There was a significant linear effect in depression symptoms (β=–.49 t_99.24_=–20.64, *P*<.001), indicating a .49 decrease in depression symptoms with each week in care. The quadratic effect was also significant (β=.04 t_90.41_=10.72, *P*<.001), suggesting a decelerated decrease in depression symptoms over time (Table 1).

For anxiety symptoms at 6 weeks, the average length of care, the mean level of symptoms in the sample was 6.57. There was a significant linear effect in anxiety symptoms (β=–.64, t_274.62_=–22.43, *P*<.001), indicating a .64 decrease of anxiety symptoms with each week in care. The quadratic effect was also significant (β=.04, t_89.79_=12.17, *P*<.001), suggesting a decelerated decrease in anxiety symptoms over time (Table 2).

**Table 1:** Unconditional growth model for depression symptoms.

PHQ-9^a^	Estimate	SE	*t*	95% CI	*P* value
Intercept	5.94	.20	28.34	5.53 to 6.36	<.001
Slope	–.49	.02	-20.64	–.53 to –.44	<.001
Quadratic	.04	.004	10.72	.03 to .05	<.001

^a^ Patient Health Questionnaire-9

**Table 2:** Unconditional growth model for anxiety symptoms.

GAD-7^a^	Estimate	SE	*t*	95% CI	*P* value
Intercept	6.57	.19	33.61	6.19 to 6.96	<.001
Slope	–.64	.02	–22.43	–.70 to –.58	<.001
Quadratic	.04	.004	12.17	.04 to .05	<.001

^a^ Generalized Anxiety Disorder-7

Age, gender, and provider identifier were included in the models for anxiety and depression symptoms as level-2 predictors of the intercept, slope, and quadratic slope in order to determine whether age, gender, and providers moderated the outcomes for participants. Likelihood ratio testing revealed a better fit for models including age, gender, and provider identifiers as level-2 predictors of the intercept only, thus that model was retained. For both depression and anxiety symptoms, neither the age, gender, or provider were statistically significant, suggesting that the intercept, slope, and quadratic slope were not related to these variables (Tables 3 and 4). 

**Table 3:** Age, gender, and provider as predictors of the intercept, slope, and quadratic components for depression symptoms.

PHQ-9^a^	Estimate	SE	*t*	95% CI	*P* value
Intercept	6.51	.87	7.48	4.80 to 8.22	<.001
Slope	–.58	.02	–20.53	–.63 to –.52	<.001
Quadratic	.04	.004	10.72	.03 to .05	<.001
Age	–.01	.02	–.67	–.05 to .02	.49
Gender	.18	.37	.48	–.55 to .91	.62
Provider	–.007	.01	–.43	–.04 to .02	.66

^a^ Patient Health Questionnaire-9

**Table 4:** Age, gender, and provider as predictors of the intercept, slope, and quadratic components for anxiety symptoms.

GAD-7^a^	Estimate	SE	t	95% CI	p-value
Intercept	6.75	.78	8.63	5.21 - 8.29	<.001
Slope	-.64	.02	-22.25	-.70 - -.58	<.001
Quadratic	.04	.003	12.10	.04 - .05	<.001
Age	-.002	.02	-.14	-.04 - .03	.88
Gender	.28	.33	.85	-.37 - .94	.39
Provider	-.01	.01	-1.01	-.04 - .01	.31

^a^ Generalized Anxiety Disorder-7

### Paired samples t-tests

#### Participants Starting With Depression or Anxiety Symptoms in Clinical Range

The mean (SD) scores of pre-treatment GAD-7 across all participants with baseline clinical range of depression or anxiety symptoms (n=385) was 11.72 (3.89) and of PHQ-9 was 10.77 (4.71), corresponding to moderate anxiety and moderate depression (Table 5).

**Table 5:** Changes in depression and anxiety symptoms for participants starting at clinical levels of depression or anxiety symptoms (n=385).

Measure	Baseline score, mean (SD)	Follow-up score, mean (SD)	Paired differences, mean (SD)	95% CI of the difference	t-value (*df*)	*P* value	Cohen *d*
PHQ-9^a^	10.77 (4.71)	5.57 (4.92)	5.20 (5.52)	4.52-5.88	18.49 (384)	<.001	1.08
GAD-7^b^	11.72 (3.89)	6.07 (4.55)	5.65 (5.40)	5.06-6.25	20.55 (384)	<.001	1.33

^a^ Patient Health Questionnaire-9

^b^ Generalized Anxiety Disorder-7

Reliable clinical change on either the PHQ-9 and/or GAD-7 measures was observed in 283 participants (73.5%), 319 participants (82.8%) recovered on either measure, and 258 participants (67%) demonstrated clinically significant improvement in either depression or anxiety (Table 6).

**Table 6:** Reliable improvement and recovery for participants grouped by their baseline levels of depression and anxiety symptoms.

Participant sub-group	Reliable improvement, n (%)	Recovery, n (%)	Reliable improvement and recovery, n (%)	Reliable improvement or recovery, n (%)
Baseline depression (PHQ-9 ≥10; n=225)	149 (66.2)	167 (74.2)	141 (62.7)	175 (77.8)
Baseline anxiety (GAD-7≥8; n=352)	245 (69.6)	247 (70.2)	217 (61.7)	275 (78.1)
Baseline depression and anxiety (PHQ-9≥10 and GAD-7≥8; n=192)	115 (59.8)^a^	120 (62.5)^b^	105 (54.6)^c^	130 (67.7)^d^
Baseline depression or anxiety (PHQ-9≥10 or GAD-7≥8; n=385)	283 (73.5)^e^	319 (82.8)^f^	258 (67.0)^g^	336 (87.2)^h^

^a^ Calculated as reliable improvement on PHQ-9 AND GAD-7

^b^ Calculated as recovery on PHQ-9 AND GAD-7

^c^ Calculated as (reliable improvement and recovery on PHQ-9) OR (reliable improvement and recovery on GAD-7)

^d^ Calculated as (reliable improvement OR recovery on PHQ9) OR (reliable improvement OR recovery on GAD7)

^e^ Calculated as reliable improvement on PHQ-9 OR GAD-7

^f^ Calculated as recovery on PHQ-9 OR GAD-7

^g^ Calculated as (reliable improvement AND recovery on PHQ-9) OR (reliable improvement AND recovery on GAD-7)

^h^ Calculated as (reliable improvement OR recovery on PHQ-9) OR (reliable improvement OR recovery on GAD-7)

#### Participants Starting With Depression Symptoms in Clinical Range

At pre-treatment, 225 participants (58.4%) scored in the clinical range on the PHQ-9. The mean of the pre-treatment PHQ-9 score was 13.88 (SD 3.40), corresponding to moderate depression, while the post-treatment score was 6.76 (SD 5.35). Results of paired samples *t-*tests revealed that for this group, depression scores decreased significantly, with an average reduction of 7.12 (SD 5.74) points, (t_224_=18.62, *P*<.001, Cohen *d*=1.59), suggesting a large effect of treatment on depression symptoms.

Reliable clinical change in depression scores was observed in 149 participants (66.2%), and 167 participants (74.2%) recovered on the PHQ-9. A total of 141 participants (62.7%) demonstrated clinically significant improvement on the PHQ-9, ie, meeting criteria for both reliable clinical change and recovery (Table 6).

#### Participants Starting With Anxiety Symptoms in Clinical Range

At pre-treatment, 352 participants (91.4%) scored in the clinical range on the GAD-7. The mean of pre-treatment GAD-7 score for this group was 12.32 (SD 3.49), corresponding to moderate anxiety, while the post-treatment score was 6.24 (SD 4.56).

Anxiety scores decreased significantly by an average of 6.08 (SD 5.31) points, (t_351_=21.47, *P*<.001, Cohen *d*=1.50), suggesting a substantial effect of treatment on anxiety. Reliable clinical change was observed in 245 (69.6%) participants, 247 participants (70.2%) recovered, and 217 (61.7%) participants demonstrated clinically significant improvement (Table 5).

#### Participants Starting With Depression and Anxiety Symptoms in Clinical Range

A total of 192 participants (49.9%) had pre-treatment PHQ-9 and GAD-7 scores in the clinical range, suggesting comorbid symptoms for both depression and anxiety. In this group, depression scores decreased significantly by an average of 7.13 (SD 5.93) points, (t_191_=16.67, *P*<.001, Cohen *d*=1.56), and anxiety by an average of 6.79 (SD 5.92) points, (t_191_=15.88, *P*<.001, Cohen *d*=1.55), suggesting large and similar effects of treatment on both anxiety and depression (Table 7).

Reliable clinical change in both measures was observed for 115 participants (59.8%), 120 participants (62.5%) recovered on both measures, and 105 participants (54.6%) demonstrated clinically significant improvement in both depression and anxiety (Table 6). 

**Table 7:** Changes in depression and anxiety symptoms for participants starting at clinical levels of depression and anxiety symptoms (n=192).

Measure	Baseline score (SD)	Follow-up score (SD)	Paired differences mean (SD)	95% CI of the difference	*t* value (df)	*P* value	Cohen *d*
PHQ-9^a^	14.14 (3.49)	7.01 (5.43)	7.13 (5.93)	6.21-8.05	16.67 (191)	<.001	1.56
GAD-7^b^	13.60 (3.61)	6.82 (5.04)	6.79 (5.92)	5.91-7.67	15.88 (191)	<.001	1.55

^a^ Patient Health Questionnaire-9

^b^ Generalized Anxiety Disorder-7

## Discussion

To our knowledge, this is the first study to examine the effectiveness of BC-CBT delivered via video at a large scale in a real-world setting within the US. The BC-CBT program evaluated combined live video-based sessions with a therapist plus technology-based care tools that clients had access to and were encouraged to use in between sessions. Our results suggest that BC-CBT can be effective in significantly reducing symptoms of depression and anxiety. Age, gender, and specific psychotherapists did not moderate the changes in symptoms observed. The within-subjects effect sizes were similar and similarly large for both depression (*d*=1.59) and anxiety (*d*=1.50) symptom improvements for individuals starting at clinical levels of depression and anxiety symptoms at baseline. These results were similar to those reported in various randomized trials for adult depression [47] and similar to those reported for effectiveness trials of adult anxiety disorders [48]. Notably, these results were observed after treatment lasting an average of 5.2 sessions and 6.4 weeks, which is significantly shorter than the conventional 12 to 16 weekly sessions protocol of most CBT treatments [49,50]. Outcomes collected more than 2 weeks before the first therapy session or more than 5 weeks after the last therapy session were considered invalid. If baseline outcomes were collected too early, or too much time had passed between the end of care and the last assessment, such data likely no longer reflected actual symptoms at the beginning and end of care. Our findings suggest that the BC-CBT program and its intense psychotherapy experience, resulting from combining video therapy sessions with personalized digital tools, were successful in delivering a clinically efficient treatment, as demonstrated by participants making great progress in a short duration of time. Thus, CBT can be delivered via alternative modalities, ie, via video and blended with technologically based approaches, in an ecologically valid manner, and remain both effective and efficient.

An essential component of CBT is engaging in homework between therapy sessions. Indeed, homework compliance has been consistently associated with better outcomes for CBT [51,52], yet compliance is commonly low [53]. In our BC-CBT program, therapists assigned homework such as digital lessons and exercises through a digital platform, which enabled them to monitor whether and when the participant did the homework. If the participant struggled or procrastinated to complete the homework, the therapist could send a personalized reminder and encouragement about the homework, which could increase the client’s motivation and compliance. The combination of individual provider-led therapy sessions with personalized digital components to foster consolidation of CBT skills allowed clients to receive personalized care and encouragement for increased treatment engagement and completion. Future research should investigate the specific impact of digital homework such as digital lessons and exercises, and personalized messages from the provider to encourage homework completion, on the effectiveness and efficacy of the BC-CBT treatment.

This study has several limitations. We did not utilize an RCT design, and our results should thus be interpreted with caution. Although an RCT design might offer a more precise understanding regarding the effectiveness of a treatment, randomization is not feasible in a setting where care is offered as an employer-sponsored benefit given ethical complexities related to limiting care options for participants. Given that no control group was included, we cannot be entirely certain that the results reported are indeed due to the BC-CBT intervention rather than due to the passage of time or other factors. However, comparing our outcomes to well-conducted RCTs, we have observed that our outcomes are comparable, lending support for the validity of our results. Another study limitation is that though we assessed for meaningful change by calculating reliable clinical change, recovery, and clinically significant change, whether symptom improvement was maintained in the long-term is not known. Participants were either employees or dependents of employed individuals, mostly younger, and without symptoms of severe mental illness or acute suicidality, or homicidality; thus, the results may not generalize to other populations or those with acute safety concerns. Outcomes were based on self-reported measures, which can be less reliable in assessing levels of symptoms compared to clinician-administered interviews due to possible misinterpretation of the questions by clients and the lack of follow-up clarifying questions from the provider.

In conclusion, this study demonstrates that BC-CBT can effectively reduce depressive and anxiety symptoms outside of the clinical trial environment. Future research should examine the separate and synergistic contributions of the therapist-led versus the digital components of care to treatment outcomes. Having a better understanding of the optimal ratio of the two modalities, as well as the matching of this ratio to client-specific variables in order to maximize treatment outcomes for different participants and presenting issues, will allow us to enhance and personalize BC-CBT.

## Appendix

Multimedia Appendix 1 Sample BC-CBT components: secure messaging, video therapy, and digital lessons.

## Abbreviations

BC-CBT: blended care cognitive behavioral therapy
CBT: cognitive-behavioral therapy
GAD-7: Generalize Anxiety Disorder-7
HIPAA: Health Insurance Portability and Accountability Act
HLM: hierarchical linear modeling
iCBT: internet-administered automated cognitive behavior therapy
PHQ: Patient Health Questionnaire-9
RCT: randomized controlled trial
