# Supplementary figures and images for: Blended Care-Cognitive Behavioral Therapy for Depression and Anxiety in Real-World Settings: Pragmatic Retrospective Study
Source: J Med Internet Res. 2020 Jul 6;22(7):e18723. doi: 10.2196/18723 (PMC7381079; doi:10.2196/18723)

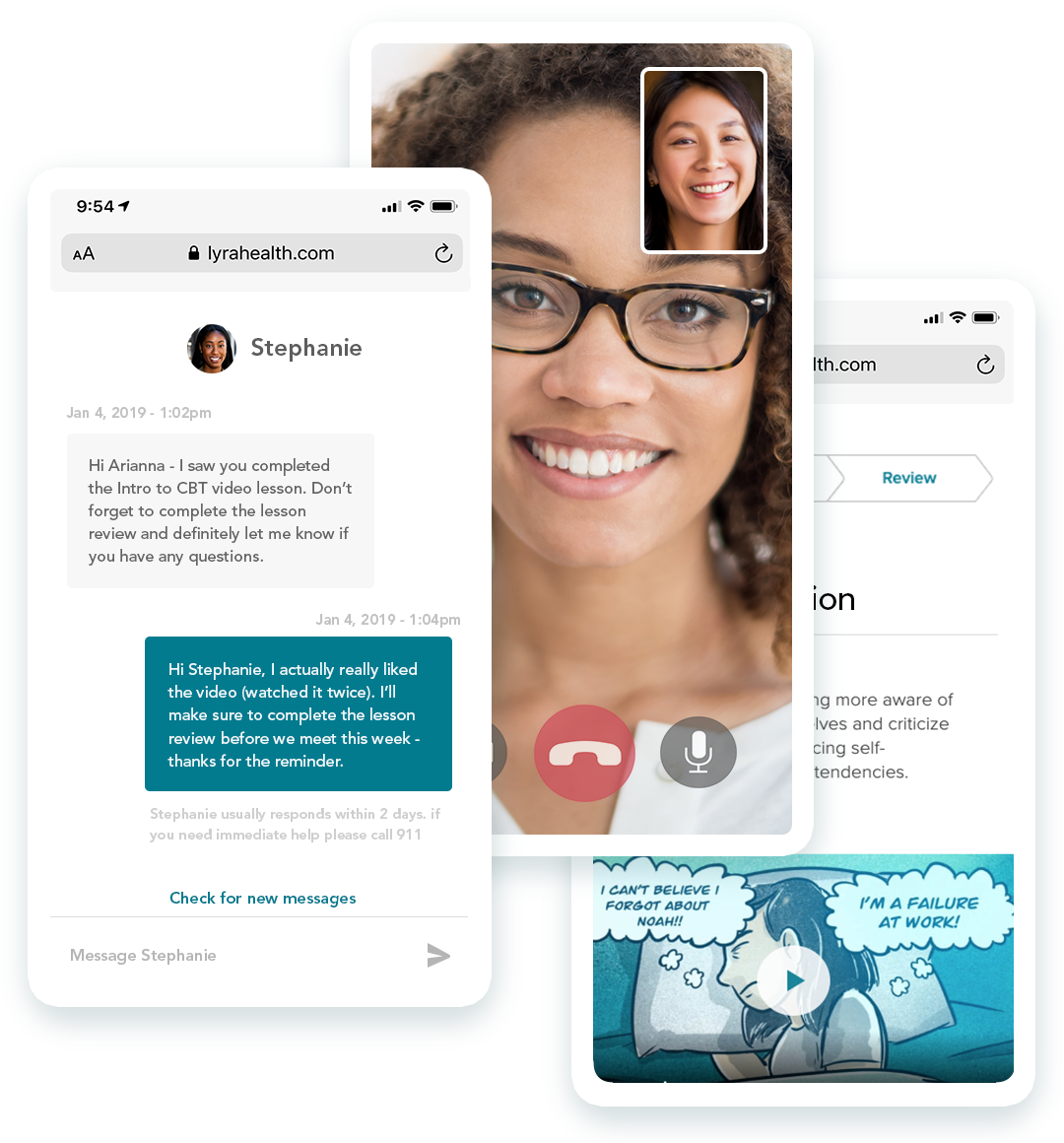

Supplement: Multimedia Appendix 1 [file jmir_v22i7e18723_app1.png]
